# Dynamic, Large-Scale Profiling of Transcription Factor Activity from Live Cells in 3D Culture

**DOI:** 10.1371/journal.pone.0014026

**Published:** 2010-11-17

**Authors:** Michael S. Weiss, Beatriz Peñalver Bernabé, Abigail D. Bellis, Linda J. Broadbelt, Jacqueline S. Jeruss, Lonnie D. Shea

**Affiliations:** 1 Department of Chemical and Biological Engineering, Northwestern University, Evanston, Illinois, United States of America; 2 Department of Surgery, Feinberg School of Medicine, Northwestern University, Chicago, Illinois, United States of America; 3 Robert H. Lurie Comprehensive Cancer Center, Northwestern University, Chicago, Illinois, United States of America; 4 Institute for Bionanotechnology in Medicine (IBNAM), Northwestern University, Chicago, Illinois, United States of America; University of Dayton, United States of America

## Abstract

**Background:**

Extracellular activation of signal transduction pathways and their downstream target transcription factors (TFs) are critical regulators of cellular processes and tissue development. The intracellular signaling network is complex, and techniques that quantify the activities of numerous pathways and connect their activities to the resulting phenotype would identify the signals and mechanisms regulating tissue development. The ability to investigate tissue development should capture the dynamic pathway activity and requires an environment that supports cellular organization into structures that mimic *in vivo* phenotypes. Taken together, our objective was to develop cellular arrays for dynamic, large-scale quantification of TF activity as cells organized into spherical structures within 3D culture.

**Methodology/Principal Findings:**

TF-specific and normalization reporter constructs were delivered in parallel to a cellular array containing a well-established breast cancer cell line cultured in Matrigel. Bioluminescence imaging provided a rapid, non-invasive, and sensitive method to quantify luciferase levels, and was applied repeatedly on each sample to monitor dynamic activity. Arrays measuring 28 TFs identified up to 19 active, with 13 factors changing significantly over time. Stimulation of cells with β-estradiol or activin A resulted in differential TF activity profiles evolving from initial stimulation of the ligand. Many TFs changed as expected based on previous reports, yet arrays were able to replicate these results in a single experiment. Additionally, arrays identified TFs that had not previously been linked with activin A.

**Conclusions/Significance:**

This system provides a method for large-scale, non-invasive, and dynamic quantification of signaling pathway activity as cells organize into structures. The arrays may find utility for investigating mechanisms regulating normal and abnormal tissue growth, biomaterial design, or as a platform for screening therapeutics.

## Introduction

Signaling pathways provide communication from the extracellular environment to direct cellular responses, and molecular defects leading to aberrant pathway activity are associated with many disease processes [1], [2], [3], [4]. An extracellular stimulus, such as a growth factor binding to a receptor, initiates a cascade of reactions that may terminate in the activation of a transcription factor (TF) that influences gene expression. At any moment, a cell may experience multiple extracellular stimuli and these signals are processed by the complex intracellular network to determine the cellular response. The analysis of cellular responses to extracellular stimuli is complicated by the interconnectedness of signaling pathways, including cross-talk between pathways and redundancy of pathway activity [5], [6], [7]. A technique for large-scale, quantitative, and dynamic measurement of signaling pathway activity would be an enabling tool to investigate normal and abnormal cellular processes. Genomics and proteomics, the most common large-scale techniques, report on specific mRNA or proteins levels, and are not ideally suited to report pathway activity. Alternatively, pathway activity can be quantified through TFs, for which reporter constructs have been developed with consensus binding sites that regulate production of a reporter gene. This goal of large-scale analysis of TF activity has been performed through creation of cell lines stably expressing TF reporters [8], yet a transfection-based approach could be performed more quickly and extended to a range of cell types. Transfection has been widely used to deliver constructs that report on TF activity, though this transfection is typically performed on a focused number of pathways.

The investigation of active pathways and cellular responses performed within an environment where cells receive signals from the extracellular matrix, neighboring cells, and soluble factors within the local milieu would be an effective means of elucidating regulatory mechanisms. Natural and synthetic hydrogels are being employed to mimic the *in vivo* environment by providing a three-dimensional (3D) support for cells allowing the formation of multi-cellular structures [9]. Cellular responses in hydrogels can differ significantly from more traditional 2D cell culture. For example, normal mammary epithelial cells in 3D culture form acinar structures with hollow lumens whereas breast cancer cells form structures with multiple degrees of disorder [10], and these phenotypes are not observed on polystyrene. Additionally, 3D culture leads to a differential response to growth factors, hormones, extracellular matrix proteins, and chemotherapy drugs [11], [12]. Taken together, hydrogels for 3D culture that support the formation of complex phenotypes provide valuable tools for investigating the connection between the extracellular stimuli and the cellular response.

We report a system for rapid, non-invasive, large-scale, dynamic quantification of TF activity for cells growing in hydrogels. This system is based on the parallel delivery of TF reporter constructs to cells cultured within hydrogels in an array format. The TF constructs encode for luciferase reporters, which are highly sensitive and easily quantified to measure transcriptional activity. Bioluminescence imaging can be employed to rapidly quantify luciferase levels in a non-invasive manner [13] and can be repeated to monitor pathway activity over time. While this system is applicable for 2D culture, these assays are performed for cells cultured in hydrogels to support and recreate the 3D environment and the organization of multi-cellular structures. This system can be a central technology to quantify activity of numerous TFs and signaling pathways, which can provide a platform to screen therapeutic compounds, identify pathways with aberrant activity that could be targeted for therapy, or design environments that promote specific cellular processes in regenerative medicine.

## Results

### Growth and transfection in hydrogels

**Figure 1:**
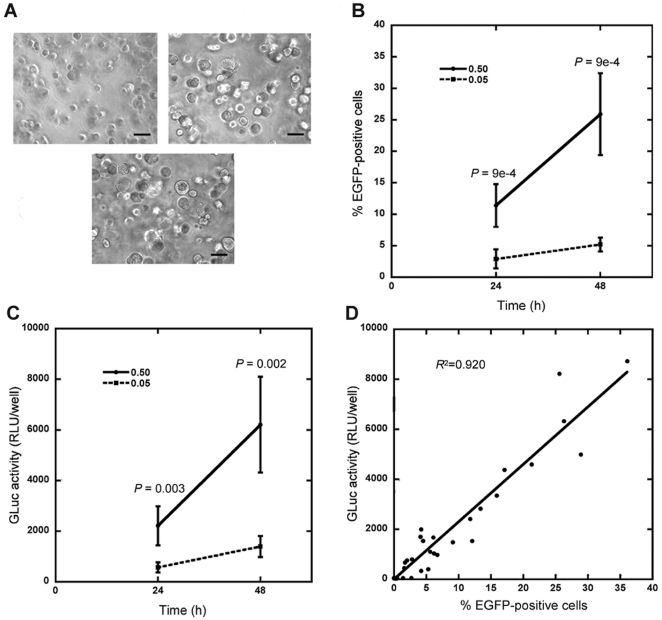
Reporter constructs to normalize for transfection efficiency during structure formation in hydrogels. (A) MCF-7/WS8 cells growing within Matrigel hydrogels without DNA complexes began as individual cells at day 1 (top left panel) and formed multi-cellular spheroids by day 3 (top right panel), a process that was not inhibited by 0.5 µg DNA lipoplexes (bottom panel). Scale bar, 200 µm. (B–D) Cells were transfected with reporter genes for EGFP and GLuc in parallel (0.5 or 0.05 µg DNA total with an pEGFP:pGLuc mass ratio of 9∶1) within hydrogels. Percentage of EGFP positive cells (B) and activity of GLuc (C) increased with DNA amount at both 24 and 48 h. Values are means ± s.d. from two independent experiments carried out in triplicate. GLuc activity correlated with %EGFP-positive cells (D).

Initial studies investigated transfection of MCF-7/WS8 cells cultured in Matrigel. This natural matrix has been used for numerous cell types [14], including MCF-7/WS8 cells, which are a well-differentiated cancerous breast cancer cell line selected for estrogen sensitivity [15]. Cells seeded inside Matrigel grew into multi-cellular spheroids during the first three days of culture (Fig. 1A), a process not perturbed by the presence of DNA lipoplexes (Fig. 1A). Enhanced green fluorescent protein (EGFP) and Gaussia luciferase (GLuc) reporter vectors were used to characterize transfection. Increasing the amount of DNA resulted in increased numbers of transfected cells (Fig. 1B) and protein production (Fig. 1C) after both time points. The outputs of EGFP and GLuc were correlated (Fig. 1D), indicating that GLuc was an appropriate measure for normalization of transfection efficiency. All subsequent studies used 0.5 µg of DNA lipoplexes, as a higher transfection efficiency was achieved.

### Reporter constructs to quantify TF activity in hydrogels

**Figure 2:**
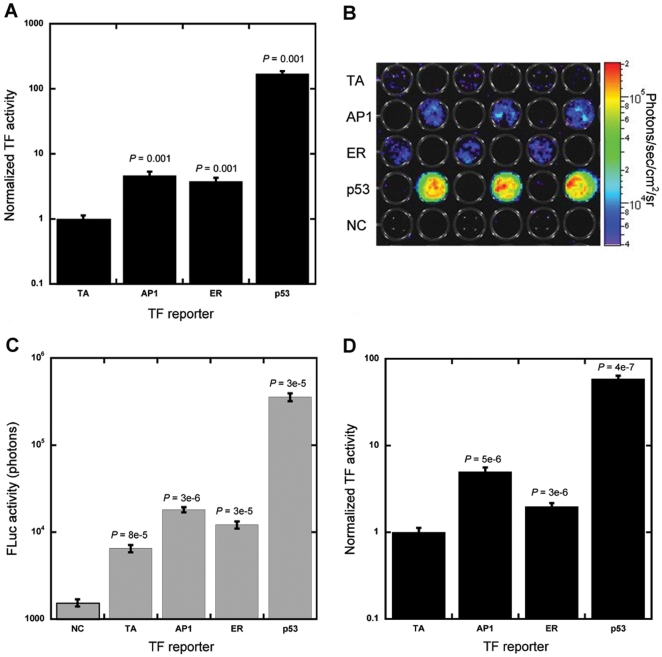
Dual-luciferase reporter constructs to measure TF activity. (A–D) MCF-7/WS8 cells within hydrogels were transfected in parallel with pFLuc reporter constructs containing enhancer elements for specific TFs (AP1, ER, and p53), vector control (TA), or not transfected (NC). For wells with reporter constructs, a constituently active pGLuc construct was delivered to normalize for transfection efficiency. (A) Cells were extracted and lysed after 48 h to determine FLuc production, which was normalized to transfection efficiency. (B–D) Bioluminescence imaging was used to non-invasively quantify FLuc production after 48 h from cells growing within Matrigel. (B) Pseudo-color mapping demonstrates localized luminescence output from cells in hydrogels seeded in alternating wells of a 96-well plate. (C) Raw FLuc signals were significantly greater than bioluminescence noise from NC. (D) FLuc signals from bioluminescence imaging were normalized to transfection efficiency and normalized TF activities for all three factors were significantly greater than TA. Values are means ± s.d. from at least three independent experiments carried out in triplicate.

Subsequent studies investigated the quantification of TF activity for cells cultured in hydrogels using a dual-construct system: i) a signal transduction reporter construct containing a TF binding site that modifies the activity of a basal promoter to produce firefly luciferase (FLuc), and ii) a normalization construct that contains a constitutive promoter driving production of GLuc. Each well of the array contained MCF-7/WS8 cells mixed with TF and normalization construct lipoplexes and then encapsulated within Matrigel. Initial studies employed three TF reporter vectors and one control vector containing only a TATA box driving FLuc expression (TA). AP1 regulates cell cycle progression and influences cell proliferation and transformation [16]. ER is a steroid receptor that regulates growth and apoptosis of cells and is a target of hormone therapy [17]. p53 is a tumor suppressor protein that can induce apoptosis upon DNA damage [18] and is involved in differentiation [19]. To quantify TF activity after 48 h, GLuc was measured in culture media and FLuc was measured from cell lysates. Normalized light emission for each sample was determined by dividing the FLuc reading by the GLuc reading and normalized TF activity was determined by dividing the normalized light emission for each sample by the average normalized light emission for the TA control. Normalizing FLuc to GLuc was used to account for differences in transfection efficiency and cell number and normalization to the TA control was applied to account for differences in TA promoter activity and GLuc accumulation. All TFs produced light emission that was significantly greater than the TA control (Fig. 2A), demonstrating reporter constructs could quantify TF activity in hydrogels. Activities were detected over multiple orders of magnitude as well; AP1 and ER were both about 4-fold above control and p53 was increased more than 100-fold.

Bioluminescence imaging was also used for rapid, non-invasive quantification of TF activity for cells growing in Matrigel. Rather than extracting and lysing cells, the substrate d-luciferin was added directly to media and light production was quantified by imaging the three TF reporters, the TA control, and non-transfected cells (NC) (Fig. 2B). Luminescence from transfected cells was significantly greater than background measured within non-transfected cells (Fig. 2C). Normalized TF activities for all three TF reporters were greater than TA (Fig. 2D), consistent with values from extracting and lysing cells. Control studies involving the sequential replacement of GLuc and FLuc vectors with a β-galactosidase vector did not affect either the GLuc or bioluminescence readings (data not shown). Bioluminescence imaging enabled rapid and non-invasive quantification of TF activity within living cells cultured within hydrogels and output was consistent with more standard extraction and lysis techniques.

### TF activity in response to β-estradiol treatment

**Figure 3:**
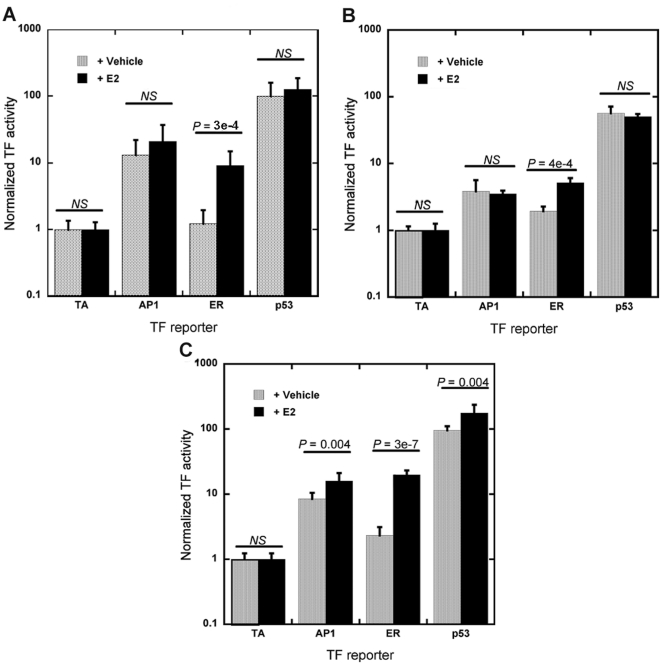
Dual-luciferase constructs to capture changes in TF activity in response to E_2_ treatment. (A,B) MCF-7/WS8 cells transfected with pFLuc and pGLuc reporter constructs and seeded within Matrigel were treated with E_2_ for 24 h. TF activity was measured by extracting cells from hydrogels and lysing (A) or by bioluminescence imaging (B). (C) MCF-7/WS8 cells were grown on 2D polystyrene, transfected, and treated with E_2_ for 24 h. TF activity was measured by lysing cells. Values are means ± s.d. from at least three independent experiments carried out in triplicate. *NS* indicates no significant difference with treatment.

The system was subsequently evaluated for its ability to detect changes in TF activity within Matrigel. Experiments were performed in the absence and presence of β-estradiol (E_2_), which was used to derive the MCF-7/WS8 cells. E_2_ signals by directly binding ER, which translocates to the nucleus and binds DNA [20], and also influences AP1 and p53 activities [21], [22]. Cells were treated with E_2_ or vehicle control for 24 hours prior to analysis, and TF activity was determined with both cell lysis (Fig. 3A) and bioluminescence imaging (Fig. 3B). In 3D culture, ER had a significant increase with E_2_ treatment but AP1 and p53 did not change with both techniques. The traditional approach of growing cells on polystyrene (2D) resulted in significant increases in AP1, ER, and p53 activities (Fig. 3C). Laminin, a major component of Matrigel, reportedly decreases MCF-7 response to E_2_ [23] and may account for differences between 2D and 3D culture. The dual-reporter constructs could capture differential TF activity within hydrogels with cell lysis as well as with bioluminescence imaging.

### Large-scale 3D transfected cell arrays

**Figure 4:**
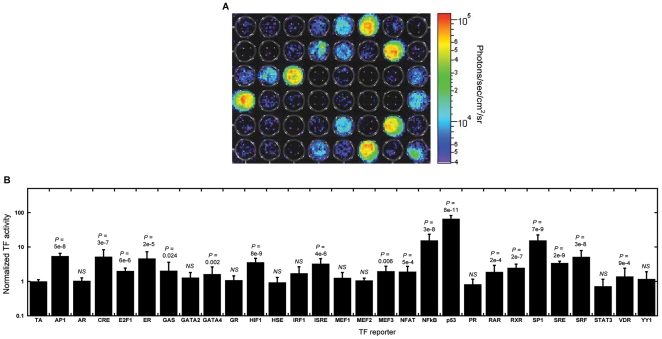
Large-scale arrays to monitor TF signaling in parallel. (A) Pseudo-color mapping indicated localized luminescence from wells throughout a portion of a 96-well plate randomly seeded with 16 conditions. (B) Quantification of TF activity at 24 h by bioluminescence imaging for 28 TFs. Values are means ± s.d. from at least three independent experiments carried out in triplicate. *NS* indicates not significantly greater than the TA control.

Arrays were created and analyzed with bioluminescence imaging to quantify 28 TFs (Fig. 4A) described in Table S1. Eighteen normalized TF activities were significantly greater than control: AP1, CRE, E2F1, ER, GAS, GATA4, HIF1, ISRE, MEF3, NFAT, NFκB, p53, RAR, RXR, SP1, SRE, SRF and VDR (Fig. 4B). Many of these TFs regulate cellular processes involved in breast cancer [6], [24], [25] and are active in MCF-7 cells [16], [26], [27], [28]. Again, luminescence quantified activities over multiple orders of magnitude. Replicates within arrays had low variability (Fig. S1) and good correlation (R^2^>0.96) (Fig. S2), with weaker correlations across arrays (0.70<R^2^<0.89). Data from the same array clustered together by Euclidian distance of the mean normalized activities (Fig. S3), suggesting blocking data by array would be appropriate for statistical analyses. A few factors were less active than TA, most noticeably STAT3. While TFs are able to act as repressors of activity [29], a repressor would decrease bioluminescence and produce values closer to background, and our analysis most effectively identified those factors that produced luminescence significantly greater than TA.

### Dyamic TF activity in response to treatment

**Figure 5:**
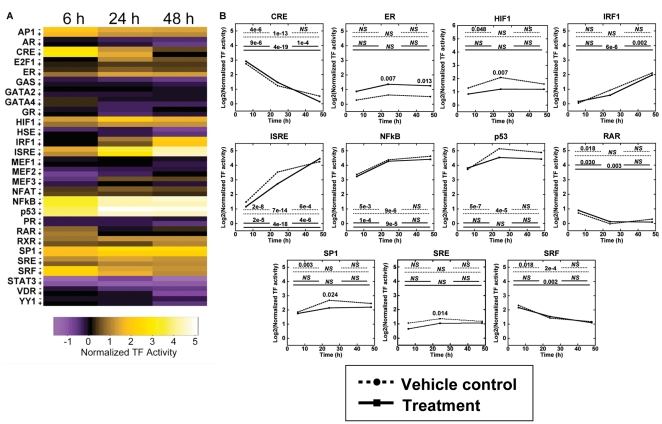
Dynamic large-scale activities of TFs in response to E_2_ treatment. (A) TFs had variable activities as functions of treatment and time, as indicated by a heat map representation of log_2_ transformation of the means. Vehicle control indicated by ‘−’ and E_2_ treatment indicated by ‘+’. (B) TFs that had significant changes with treatment or time are presented on individual temporal plots, with log_2_ transformations of the TF activity means plotted. Dashed lines indicate vehicle control and solid lines indicate E_2_ treated. Horizontal lines represent comparisons at different time points for each condition, with 6 h to 24 h (top left), 24 h to 48 h (top right) and 6 h to 48 h (bottom). *NS* indicates no significant difference. A significant difference with E_2_ treatment is indicated with the p-value at the significant time point. For example, CRE had significant differences in activity between 6 and 24 h (*P = *4e-6) and between 6 and 48 h (*P* = 1e-13) for untreated cells, and significant differences for all temporal comparisons for E_2_ treated cells. ER had significantly greater activity at 24 and 48 h (*P* = 0.007, *P* = 0.013), but no significant differences with time. Values are log_2_ transformed means from at least three independent experiments carried out in duplicate for each condition.

The 3D transfected cell arrays were subsequently employed for large scale reporting on the dynamic changes in TF activity in response to E_2_ treatment. Cells were treated with E_2_ or vehicle control, and TF activity was quantified at 6, 24, and 48 h (Fig. 5A). ER, HIF1, SP1, and SRE all showed significant changes in activity in response to treatment (Fig. 5B). The increased ER activity mirrored the response obtained with the small-scale array, and decreased HIF1, SP1, and SRE activities at 24 h indicated that E_2_ reduced induction. HIF1, IRF1, ISRE, NFκB, p53, and SP1 had activities that increased with time and CRE, RAR, and SRF had activities that decreased with time (Fig. 5B). These changes were consistent with increased cell survival and inflammatory signaling associated with cancerous growth [25], [30] as well as decreased Rho and ERK signaling associated with transferring cells to 3D culture [31], [32].

**Figure 6:**
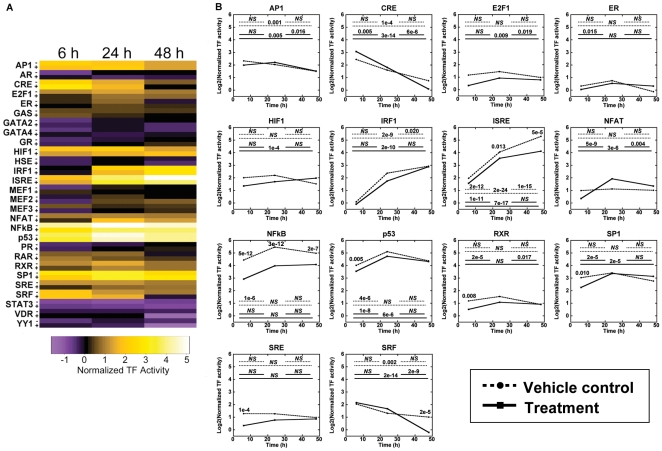
Dynamic large-scale activities of TFs in response to activin A treatment. (A) TFs had variable activities as functions of treatment and time, as indicated by a heat map representation of log_2_ transformation of the means. Vehicle control indicated by ‘−’ and activin A treatment indicated by ‘+’. (B) TFs that had significant changes with treatment or time are presented on individual temporal plots, with log_2_ transformations of the TF activity means plotted. Dashed lines represent vehicle control and solid lines represent activin A treatment. Statistical comparisons are described in legend of Figure 5. Values are log_2_ transformed means from at least three independent experiments carried out in duplicate for each condition.

Activin A treatment was also investigated to identify differential TF profiles in response to another factor that influences cancer progression. Activin A belongs to the TGF-β superfamily and acts via Smad signaling [33]. FLuc and GLuc were measured 6, 24, and 48 h after treatment with activin A or vehicle. Heat map representation of TF activity demonstrated dynamic activity of numerous TFs (Fig. 6A) with significant differences (Fig. 6B). Activin A induced early decreases in NFκB, p53, RXR, SP1, and SRE activities, and later decreases in ISRE and SRF activities. These changes are associated with decreased cell survival [30] and increased differentiation [34]. Eight of the nine TFs that had temporal changes in the E_2_ experiments had similar changes following stimulation with activin A, yet five additional TFs had significant changes: AP1, E2F1, ER, NFAT, and RXR. Of these five TFs, AP1 was the only factor to change over time with vehicle control. Using both E_2_ and activin A treatments, differential and dynamic TF activities were measured with the 3D transfected cell arrays, demonstrating the system can capture widespread signaling as cells organize into multi-cellular structures.

## Discussion

Signal transduction pathways that terminate in TF activation are non-linear, with cross-talk at multiple levels that affects activity across pathways [5], [6], [7]. We report a method for rapid, dynamic, and large-scale quantification of TF activity for cells cultured in hydrogels that support cellular organization into multi-cellular structures. This cellular array analyzed the activity of 28 TFs, and at least 10 of the 28 TFs had activity that varied as a function of time or biochemical stimulation. The implementation of bioluminescence imaging for luciferase reporters is a direct measure of transcriptional activity, which contrasts with the large-scale genomics and proteomics approaches that are more typically employed for pathway analysis [35], [36]. These more standard approaches assume that the presence of a pathway component implies its activity, yet they do not capture the impact of phosphorylation state or cellular compartmentalization that are common mechanisms for regulating pathway activity. Phosphorylation, as in the MAP kinase pathway, is a common mechanism to specifically regulate protein-protein interactions within the pathway, and TF binding to DNA may depend on its phosphorylation state [37]. Compartmentalization similarly regulates molecular interactions by segregating the factors, which is exemplified by steroid hormone receptors that are located outside of the nucleus in their inactive state, yet contact with the ligand leads to nuclear translocation and DNA binding [20], [38]. The cell arrays reported herein use living cells and thus maintain the physiological context of the intracellular network; the production of the reporter gene occurs only if the TF and all upstream components in the pathway are present, located in the appropriate cellular compartment, and have been post-translationally modified to transmit the signal.

For our system reported herein, repeated measurement of the sample allowed for longitudinal analysis of pathway activity during the development of multi-cellular structures. Cellular signaling networks are not stagnant, and signaling events can be transient or cyclical [39], [40]. Cellular responses are dependent on both the specific TFs that are active and the timing of their activities [41], [42], [43]. This TF activity array provides a large-scale view of active pathways within a system at multiple time points. Signaling pathway dynamics are typically captured on the time scale of seconds to minutes to investigate the mechanistic interactions of pathway components. This TF activity array focused on the time scales relevant to the organization of cells into structures (i.e., hours to days), and thus captured the net effect of the individual interactions that sequentially activated pathways during tissue formation or the cascade of pathways activated by an external stimulus. Expression resulting from transient transfection persists for several days, with the decreasing expression the result of degradation or silencing mechanisms. While techniques such as stable transfection or infection could extend the duration of expression, transient transfection results in substantial numbers of plasmid copies within cell nuclei within hours [44], which enables measurement of TF activity soon after cell encapsulation for analysis of the early tissue development. Transient transfection can also be performed more quickly than stable transfection and is more amenable to scale-up. Using a well-established breast cancer cell line, this array detected differential activities in processes associated with the growth of cancer cells, such as cell survival (HIF1, NFκB), proliferation (E2F1, ER, p53, RAR, RXR, SP1, SRE), differentiation (p53, SP1), invasion (NFAT), hypoxia (HIF1), and inflammation (IRF1, ISRE, NFκB) [6], [19], [24], [25], [26], [28], [45], [46]. ER, HIF1, SP1, and SRE all showed significant responses to E_2_ treatment (Fig. 5). ER activity increased, as expected, and the response persisted into later time points. However HIF1, SP1, and SRE had decreased activity relative to untreated cells only at 24 h, suggesting that E_2_ attenuated the activity of these factors at the early stages of culture. For studies performed with activin A stimulation, SP1, SRE, NFκB, RXR, and p53 had differential activity relative to vehicle at the initial study time point (Fig. 6). Decreased activity of ISRE and SRF was observed after the initial time point, suggesting these TFs respond downstream of the initial stimulation. A dynamic response of SP1, p53, and SRF in association with activin A and Smad signaling has been described [47], [48], [49] whereas the responses of SRE, NFκB, ISRE, and RXR have not been widely reported, which illustrates the power of the array to simultaneously report on known pathways and to identify unexpected connectivity of the network. Taken together, the arrays provided a large-scale identification of the cascade of signals occurring across multiple days that drive the formation of multi-cellular structures.

By using hydrogels for cell culture within the array, signal transduction pathway activity was analyzed within an environmental context that allowed for cell-cell and cell-matrix interactions that produced structures. Matrigel is often used to study the growth of mammary epithelial cells with formation of either normal (e.g. polarized) or cancerous (e.g. invasive) characteristics that differs from the monolayer formation observed on 2D polystyrene [50], [51], [52]. Morphological differences between 2D and 3D are associated with differential signaling [11], [53], including differential response to E_2_ reported herein (Fig. 3) and decreased Rho and ERK activities resulting from decreased focal adhesions and tensional forces [31], [32], [54]. AP1, CRE, and SRE had decreased activities over time and are downstream components of the MAPK pathway [55], [56], [57], which is influenced by Rho and ERK signaling [58]. Hence, our results are consistent with decreased MAPK signaling for 3D relative to 2D culture. In addition to differences in growth, cells within 3D matrices may have an *in vivo* phenotypic response to chemotherapeutics [11], [12], further supporting the value of 3D culture for analysis of cellular processes.

Bioluminescence imaging was instrumental in enabling large-scale, dynamic, quantitative measurement of TF activity. Results from bioluminescence imaging were consistent with the typical approach of cell extraction and lysis, a labor-intensive process that increased sample-to-sample variability. The normalization construct accounted for spot-to-spot variability in transfection, a variable that would otherwise hinder statistical analyses. Also, normalizing to the TA control construct was important to account for differences in transgene expression due to degradation and silencing of the reporter plasmids. Luciferase reporters provided a sensitive method due to enzymatic signal amplification, with signals detected over several orders of magnitude [59]. Dynamic analysis of TF activity in a single sample is more common with fluorescent protein reporters [8], which can be quantified by plate readers. The fluorescent reporters, however, lack signal amplification and thus have more limited detection of weak signals. Combining luciferase reporters with bioluminescence imaging provided both sensitivity and dynamic analysis, and captured the activity of numerous TFs simultaneously while minimizing the number of samples and reagents.

Our 3D TF activity array profiled the activity of numerous signaling pathways simultaneously as cells organized into structures and responded to biochemical stimuli. Bioluminescence imaging was employed as a non-destructive technique capable of repeated measurement that enabled dynamic activity to be quantified. The array detected active TFs in at least 10 of the 28 pathways profiled, and the outputs of the array were consistent and reproducible. Importantly, this activity was captured dynamically, which identified pathways that were initially activated by a biochemical stimulus, and those pathways whose activity was altered subsequent to the initial stimulus. While the TF arrays presented are applicable to 2D cultures as well, cellular behaviors in 3D can be more relevant for recapitulating phenotypes observed *in vivo* and provided the basis for establishing these 3D cell-based arrays. This tool may be an enabling technology to identify signaling events that are associated with normal and pathological growth within the context of 3D microenvironments. The array can be employed as a platform in numerous applications, such as screening therapeutic compounds, identifying pathways with aberrant activity that could be targeted for therapy, or designing environments that promote specific cellular processes in regenerative medicine.

## Materials and Methods

### Cell maintenance

MCF-7/WS8 cells (provided by V. C. Jordan, Fox Chase Cancer Center, Philadelphia, PA, USA) were maintained in RPMI-1640 media supplemented with 10% fetal bovine serum (FBS), 100 µM non-essential amino acids, 100 U antibiotic/antimycotic, 2 mM L-glutamine (Invitrogen, Carlsbad, CA, USA) and 6 ng/ml insulin (Sigma-Aldrich, St. Louis, MO, USA), and cultured at 37°C and 5% CO_2_ in a humidified incubator. For studies using E_2_ or activin A treatment, cells were maintained in phenol red-free RPMI-1640 media with 10% charcoal-stripped FBS (Invitrogen) for three days prior to seeding.

### Plasmids

Plasmids were amplified with DH5α (Invitrogen), purified using Qiagen (Valencia, CA, USA) reagents, and stored at −20°C in Tris-EDTA buffer solution (10 mM Tris, 1 mM EDTA, pH 7.4). pEGFP encoded EGFP with a CMV promoter (Clontech, Mountain View, CA, USA). pGLuc encoded GLuc with a CMV promoter (New England Biolabs, Ipswich, MA, USA). pFLuc TF reporter constructs encoded FLuc with a minimal TATA box from the herpes simplex virus with one or multiple enhancer elements upstream of the promoter that were specific for a particular TF (Panomics, Fremont, CA, USA). pFLuc TF reporter constructs were previously validated as described by the manufacturer [60]. Twenty-nine different pFLuc TF reporter plasmids were used: 28 for specific TFs and one that had no enhancer element and reported basal FLuc expression (TA).

### Transfection and 3D culture

pFLuc TF reporter constructs, or pEGFP, and pGLuc (9∶1 µg pFLuc or pEGFP: µg pGLuc) were complexed with Transfast (Promega, Madison, WI, USA) at a charge ratio of 0.5∶1.0 Transfast:DNA in Opti-MEM (Invitrogen) and incubated for 10 minutes. MCF-7/WS8 cells were centrifuged, re-suspended with DNA complexes, and incubated for 30 minutes. Cells and complexes were mixed with four times the volume of 4°C phenol red-free, growth factor-reduced Matrigel (Trevigen, Gaithersburg, MD, USA) and 50 µL of solution containing 50,000 cells and 0.5 µg total DNA was deposited in black 96-well plates (Greiner Bio-One, Monroe, NC, USA). Hydrogels were incubated at 37°C for 45 minutes and fully supplemented RPMI-1640 media was subsequently added.

Arrays of hydrogels were constructed for simultaneous reporting of TF activities. In parallel, lipoplex solutions with different pFLuc TF reporter plasmids were made, added to cells, and combined with precursor hydrogel solutions. A minimum of three hydrogels for each condition was seeded in all experiments, except in the longitudinal study in which hydrogels were seeded in duplicate. For studies that report on 28 TFs, 15 or 16 reporter constructs in triplicate were used at a time (14–15 TF reporters and 1 TA). Experiments were independently performed at least three times.

For treatment studies, culturing media was replaced after 24 hours of culture with media containing 10^−10^ M E_2_ (Sigma-Aldrich) and ethanol or 10 ng/ml activin A (gift of T. K. Woodruff, Northwestern University, Evanston, IL, USA) and Tris. Stock solutions of 20 µM E_2_ was made in 100% ethanol and stored at −20°C and 75 µg/mL activin A made in 50 mM Tris and stored at −80°C.

### Quantification of constituently active reporter genes

Transfection efficiency was determined by delivering pEGFP followed by staining with Hoescht 33258 (Invitrogen) [61]. Total numbers of fluorescent cells on each channel were counted using ImageJ. Transgene expression with GLuc was determined with 20 µL of culture media, which was measured using a GLuc assay kit (New England Biolabs) and luminometer (Turner Biosystems, Sunnyvale, CA, USA).

### Luminescence assays for FLuc activity

FLuc activity in cell lysates was quantified by extracting cells from Matrigel using a 3D cell harvesting kit (Trevigen). Cell lysates were frozen, defrosted, and subjected to a standard FLuc assay (Promega) and a luminometer.

Bioluminescence imaging was performed with an IVIS imaging system (Caliper, Hopkinton, MA, USA) as described previously [13] with minor modifications. The substrate for FLuc, d-luciferin (Caliper), was added to wells at 1 mM, incubated for 3 minutes and then imaged for 1 minute. For longitudinal studies, hydrogels were imaged, washed once with PBS, and then fresh media was replaced. Normalized light emission was determined by dividing the FLuc reading by the GLuc reading and normalized TF activity was determined by dividing the normalized light emission for each sample by the average normalized light emission for TA.

### Statistics

Data were analyzed using R [62]. For the validation studies, comparisons were performed using Welch t-tests and p-values were adjusted with the false discovery reduction (FDR) technique [63] to account for multiple comparisons (α = 0.05, significant level). For large-scale arrays, the package ‘limma’ [64] was used with base 2 logarithmic transformations on normalized data. Welch t-tests were performed to compare transformed normalized TF activity with TA within the same array and a modified meta-analysis method [65] was used to combine results from multiple arrays. Final p-values were adjusted with FDR (α = 0.05). For dynamic array experiments, normalized TF activities were base 2 logarithmic transformed and arrays were considered independently from each other and analyzed using a Parametrical Hierarchical Bayesian mode (PHBM) [66] with empirical hierarchical hyperparameters [67] to compare the effects of time and treatments on TF activities. The t-test results from independent arrays were then combined by the modified meta-analysis and adjusted by FDR as before. PHBM was also employed to determine the similarity between experiments.

## Supporting Information

Table S1 TF reporters used in large-scale transfected cell arrays.
(0.05 MB DOC)

Figure S1 Box plot of replicates from large-scale arrays. Means of log_2_ transformed normalized TF activities from three replicates across three arrays are shown. ‘1.1’ denotes array 1, replicate 1 and ‘2.3’ denotes array 2, replicate 3, etc. Means and standard deviations were consistent within arrays, with some variability between arrays. No outliers were present.
(0.05 MB DOC)

Figure S2 Pairwise comparisons of replicates from large-scale arrays. Means of log_2_ transformed normalized TF activities from three replicates across three arrays were compared. The correlation factor is represented in the bottom right corner of each graph. Comparisons within arrays showed good correlation (R^2^>0.96), and correlations across arrays were weaker (0.70<R^2^<0.89).
(0.11 MB DOC)

Figure S3 Euclidian clustering of large-scale array data. Means of log_2_ transformed normalized TF activities from three replicates across three arrays were clustered. Data are denoted as described in Fig. S1. Replicates within arrays clustered together, indicating data could be blocked by array.
(0.04 MB DOC)
