# Supplementary material for: Dynamic, Large-Scale Profiling of Transcription Factor Activity from Live Cells in 3D Culture
Source: PLoS One. 2010 Nov 17;5(11):e14026. doi: 10.1371/journal.pone.0014026 (PMC2984444; doi:10.1371/journal.pone.0014026)
Supplement: Table S1 — TF reporters used in large-scale transfected cell arrays. (0.05 MB DOC) [file pone.0014026.s001.doc]

| TF Reporter | Description of Pathway Induction | Biological Functions |
| --- | --- | --- |
| AP1 | PKC and MAPK signaling | Cell cycle |
| AR | Androgen receptor activation | Cell cycle, apoptosis |
| CRE | cAMP binding protein activation and JNK, p38, and PKA signaling | Survival, cell cycle, differentiation |
| E2F1 | Retinoblastoma-mediated cell cycle progression | Cell cycle, proliferation |
| ER | Estrogen response element activation | Proliferation, transformation |
| GAS | Signal transducer and activator of transcription 1 (STAT1) activation | Apoptosis, differentiation, inflammation |
| GATA2 | GATA binding protein 2 (GATA2) activation | Differentiation, survival |
| GATA4 | GATA4 activation | Differentiation, apoptosis |
| GR | Glucocorticoid response element activation | Cell cycle, transformation |
| HIF1 | Hypoxia response element activation | Survival, angiogenesis, differentiation |
| HSE | Heat shock factor activation | Apoptosis |
| IRF1 | Interferon regulatory factor 1 activation | Differentiation, inflammation |
| ISRE | Interferon stimulated response element activation and Jak/STAT signaling | Survival, inflammation |
| IRF1 | Interferon regulatory factor 1 activation | Differentiation, inflammation |
| MEF1 | Myogenic factor 3 activation | Differentiation, cell cycle |
| MEF2 | MADS box activation | Apoptosis |
| MEF3 | Myelin gene expression factor 3 activation | Proliferation, migration, differentiation |
| NFAT | Nuclear factor of activated T-cells signaling | Differentiation, transformation |
| NFκB | Nuclear factor κB signaling | Survival, differentiation, inflammation |
| p53 | p53 signaling | Apoptosis, transformation |
| PR | Progesterone receptor activation | Cell cycle, survival |
| RAR | Retinoic acid receptor activation | Differentiation, apoptosis |
| RXR | Reinoid X receptor activation | Apoptosis, differentiation |
| SP1 | Sp1 signaling | EMT, cell cycle, differentiation |
| SRE | Serum response element activation | Differentiation, apoptosis |
| SRF | Serum response factor activation | Differentiation, migration, apoptosis |
| STAT3 | STAT3 activation | Transformation, survival, migration |
| VDR | Vitamin D receptor activation | Apoptosis, differentiation |
