# Supplementary figures and images for: Dynamic, Large-Scale Profiling of Transcription Factor Activity from Live Cells in 3D Culture
Source: PLoS One. 2010 Nov 17;5(11):e14026. doi: 10.1371/journal.pone.0014026 (PMC2984444; doi:10.1371/journal.pone.0014026)

**
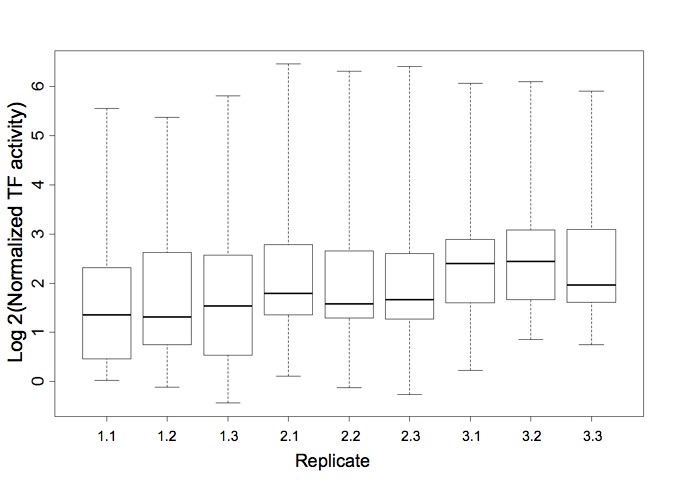
**

Supplement: Figure S1 — Box plot of replicates from large-scale arrays. Means of log2 transformed normalized TF activities from three replicates across three arrays are shown. ‘1.1’ denotes array 1, replicate 1 and ‘2.3’ denotes array 2, replicate 3, etc. Means and standard deviations were consistent within arrays, with some variability between arrays. No outliers were present. (0.05 MB DOC) [file pone.0014026.s002.doc]

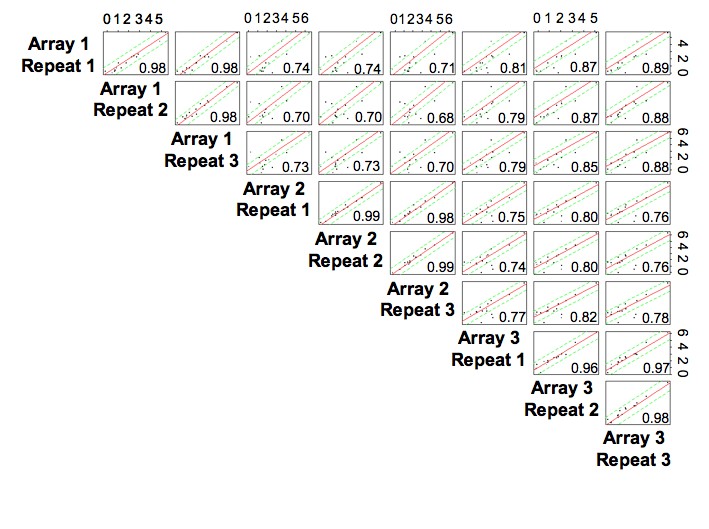

Supplement: Figure S2 — Pairwise comparisons of replicates from large-scale arrays. Means of log2 transformed normalized TF activities from three replicates across three arrays were compared. The correlation factor is represented in the bottom right corner of each graph. Comparisons within arrays showed good correlation (R2>0.96), and correlations across arrays were weaker (0.70<R2<0.89). (0.11 MB DOC) [file pone.0014026.s003.doc]

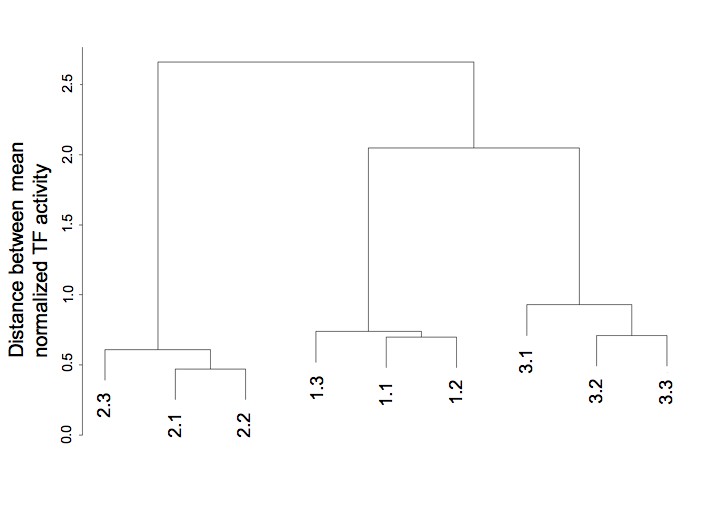

Supplement: Figure S3 — Euclidian clustering of large-scale array data. Means of log2 transformed normalized TF activities from three replicates across three arrays were clustered. Data are denoted as described in Fig. S1. Replicates within arrays clustered together, indicating data could be blocked by array. (0.04 MB DOC) [file pone.0014026.s004.doc]
